# With the 74th Regiment on the Texan Border

**Published:** 1916-10

**Authors:** H. H. Glosser

**Affiliations:** Buffalo


### With the 74th Regiment on the Texan Border.

H. H. GLOSSER, M. D., Buffalo.

Two weeks away from Buffalo. Can one have this same feeling when, roasting in the next world, he looks back longingly to that other existence before the physician signed the certificate?

Our trip down to this extreme point of southern Texas was, as the canny Scot said, "no so bad but that it might be worse no so good but that it might be better," the trip while somewhat slower than that of a letter, five days rather than three, was not more uncomfortable than one would expect in hot weather. After two nights in day coaches, sleepers were provided for the men, which, while providing room to stretch ones self comfortably, were as stuffy and ill ventilated as one could imagine.

Food was abundant and good; of the lack of variety one would soon be convinced should he mention corned beef and canned beans to any 74th man. Bread, canned tomatoes and hot coffee were also served, and on one occasion a man was seen with a can of peaches. Fresh water and ice were provided daily; while this was being done, the men detrained for exercise.

So we passed through Ohio, Indiana, Illinois, crossing the Mississippi about midnight of July 6th, and on through Missouri into Kansas. In a small town of this state we stopped for an hour and while each regaled himself in his own manner, several men sought a barber who politely but firmly closed his shop with the remark that no work was done after 8 p. m.—so the quality of patriotism varies. Such little incivilities seemed to mean much to the boys, while the slightest act of kindness brought corresponding cheer.

We experienced our first real warm weather in Oklahoma; an acquaintance once ran for governor of that state—congratulations can now be conscientiously offered on his defeat. Late one afternoon, we crossed the Red River, a dry expanse of red clay with the smallest possible stream of water; upon reaching the farther bank we found ourselves in Texas, seeing just what, according to Remington and to Collier's Magazine one would expect to see, vast tracts of grazing lands with here and there the dried skeleton of a beef critter, and what was then to us a novel sight, the cactus.

Forth Worth was reached about nightfall and for two hours the men were given such measure of freedom as they could secure while being taken through the town by their officers. It was in this town that the boys were cheered by being served with some slight refreshment by the Red Cross

Organization. All next day we travelled through Texas where one had the same feeling as on the ocean, of being a dot on the crest of infinity; the ranchers homes, simple and modest, dotted the landscape while very few tree clusters, one could not call them woods, could be seen at intervals. Always the cactus, the cattle, the irrigating ditches and the lonely homes stretched away to the horizon.

The day was Sunday and our good chaplain, John Ward, undertook the very arduous task of holding services through the train of fifteen coaches, so while we rolled over the Texan reaches, in every second car, with the aid of an impromptu choir, he held his service of scripture, song and short discourse, ending all by discussing cold lemonade in the commissary car.

About noon Houston City was reached, and the heat of Buffalo during Shriner week could have been as nothing compared to this; for two hours we walked the streets, seeking what diversion could be had in a dry city on a Sunday afternoon; after hunting in vain for a hotel with a barber shop, we decided that one might as well be converted as to live in Houston City; to the credit of the city it should be said that a post card store which the boys patronized did not charge more than the legal rate.

Entraining about the middle of the afternoon, we meandered down the line, for all the so-called fast trains were given right o'way while we sidetracked. At one stop the officials added a local freight to our twenty-five car train; all went well until a couple of violent bumps, due to trouble with the "air" and causing one officer to come into violent contact with his own heroic visage in a mirror, made the commanding officer, with the proper army language, order the extras cut out. Now we entered one of the most desolate countries imaginable; the view from the cupalo, the upstairs of the caboose, as we passed mile after mile of this wild wooded country, now and then crossing a "bayou," or creek, was more and more depressing, as the twilight advanced, until one could actually feel the yellow creeping down his back, when, at each bridge, a guard of U. S. regulars was seen to be on duty; nor were there lacking those who could tell stories of soldiers being treacherously slain by stealthy Mexicans. It was one thing to march down Main Street, between cheering crowds, another to be drawn by a powerful locomotive—alas too rapidly now—into a region where regulars were actually doing guard work. However, we did sleep that night; he was fortunate who first arose next morning and secured a bath in the last two quarts of water remaining in the Pullman. Lest someone should seize upon this point to prove the inefficiency of the army, it is necessary to add that plenty of good water was secured within an hour.

And now as the train slowed down, surely we were in the tropics, for here were date and fan palms with the proper architectural setting for all. At the next stop we were held up until a passenger train could pass, so with many misgivings we alighted and approached the station, for surely those men with big spurs, high heeled boots and guns at their hips were Texan rangers! Yes, and there were some real Mexicans! But with these rangers on guard our regiment was safe; and taking the Chaplain as protector, and with a firm grip on the pen—for has not a wise man said that the pen is mightier than the sword—we approached the country store and bought writing paper from a real Mexican, who forgot international difficulties long enough to take our cash.

At last we ceased traveling south and turned west, for now we approached our destination, Pharr, where at 4 p. m. of July 10th we detrained, marching without ceremony to the camp site, where in an incredibly short time the little shelter tents for the regiment were pitched and the men messed, i. e. ate half their remaining ration.

Now as we contemplate the comfort of our larger tents, we enjoy thinking of that first night when we slept on the ground, three in a tent built for two. As long as possible one endured mosquitoes and the language of brother officers relative thereto, then opening bed roll set up cot with mosquito bar under the dome of the midnight sky, and was falling into a pleasant slumber when a gentle rain began, one so gentle that it was hard to determine to retire, yet harder to stay; back to the tent to find that the mosquitoes had reasoned to the same conclusion; more plain talk, the refuge of the cot was again sought and enjoyed until the cruel reveille at 5.30 spoiled a pleasant morning nap.

Now began a busy day, for shelter tents were taken down to be replaced by conical tents, each of which accommodated eight men. Tents for cook shacks, each consisting of a fly, were soon occupied by the busy cooks, and long before their savory meals were ready, the conical tents were pitched in beautifully regular rows, along the company streets which extended south at regular intervals from the main camp street; to the north the officers, commissary and hospital tents were located. Latrines and shower baths were located at the outer limits of the camp; it was in the possession of these latrines and shower baths that the 74th regiment was so much more fortunate than some others in the immediate vicinity, for, with the abundant supply of water and these frame structures, the men of the 74th performed privately those duties and ablutions which others perforce did behind cheesecloth or in the open.

The first few days in camp were occupied in completing the work of erecting the tent city, for tents had to be ditched and floored, when one could secure the flooring; cook shacks must have stove foundations, refrigerators and incinerators, or brick stoves for evaporating waste water and burning solid refuse.

Now came a test of real service when the men, many of whom were taken from clerical positions, were put to work making and grading streets. It was in this trial that the men showed what was really in them, for under a tropical sun, which was rarely hidden by the merciful clouds, it was no work of light order to grade, with entrenching tools, a main street 500 feet long and fourteen company streets each averaging 200 feet in length.

The matter of feeding, always interesting, is especially so when considered in relation to large bodies of animals and men. As the child wishes to see the animals fed at the zoo, so does the interested inquirer wish to know the details of feeding en masse the soldier boy. The commissary department must see that train loads of provisions are supplied to each brigade, from which supplies the regimental commissary draws the exact amount according to the strength of the command. From the commissary each company, through its mess sergeant, draws, usually during the afternoon, one ration for each man, and woe betide that company whose cook spoils an unusual supply in the preparing. Mess call by bugle sounds at 6 a. m., 12 noon and 6 p. m., at which time the men, without the least hesitation, line up before the cook shack with meat pan, cup, knife, fork and spoon ready for first and second helping, for there is always an abundance, if the cook be a man of experience. Dishwashing disturbs the cook not at all, his part consisting of providing one boiler of hot suds, another of hot clear water, to which the men go after placing scraps of food in receptacles provided for that purpose. Garbage is carted away by enterprising Mexicans to be fed to hogs, many of which, no doubt, reach the stomachs of the men "through proper government channels."

For several weeks after arrival the men wore the woolen clothing provided at their home stations; this seemed a hardship to many people, yet it should be remembered that wool is not so uncomfortable for army men who are subject to torrential rains, and must sleep where the dews are very heavy. The lighter khaki clothing was soon provided, in which the men looked so neat it was a shame there were no white girls to see them.

To each regiment a medical corps, consisting of four medical officers, and a hospital corps, consisting of twenty enlisted men, is attached. To this medical department, styled sanitary troops, is intrusted not only the treating of the sick, but the far more important duty of keeping the camp sanitary, and the prevention of disease. To secure that end, it is the duty of the ranking medical officer, through his sanitary troops, to see that the water is potable and abundant, to provide suitable latrines built after the approved style, which must be kept free from flies by proper spraying with crude oil and carbon; to see that no tainted food is supplied and that the food eaten is properly cooked, the kitchens properly cleaned, refuse burned or carted away; he must vaccinate every man against smallpox and inject the typhoid serum. He must make a monthly examination for venereal disease, and be always on the lookout for contagious outbreaks. All this must be done in addition to treatment of the sick, which happily was very little in this camp. All the duties enumerated will be done well or ill, depending upon the ability of the ranking medical officer and his assistants. The medical officers work began at 6.20 a. m., when all who thought themselves sick reported and were passed upon, being returned to duty or placed in sick quarters, according to their real condition. If seriously ill, they were sent at once to the field hospital located three miles to the west. A report was immediately made to the commanding officer of the sick by companies, so that he knew very early in the day the exact strength of his command. At a later hour the sick reported for treatment at the infirmary tent.

There was little serious illness in the camp of the 74th regiment. The usual diarrhoeas due, no doubt, to change of water, food and climatic conditions, were rather more aggravated than was pleasant, causing the men to rise early in the morning, but there were but few serious cases. Venereal disease was surprisingly infrequent, the camps in this section being free from the usual camp followers. Catarrhal troubles were few and far between, and accidents also were rare and usually trivial.

A good word should here be said for the very excellent work done by the Y. M. C. A., which promptly upon the arrival of the troops had directors on the ground; within twelve days after the regiment arrived, the men were using the large building erected by the association, where paper, ink and envelopes were provided gratis, entertainments, both religious and secular were given, or men could sit and read quietly, there being provided a library for use in the building. Three directors were ready at all times to help the men in every way possible. Should anyone be skeptical of the good work the Y. M. C. A. is doing in the army and navy, a careful study of these buildings and these men, called to sudden duty in southern Texas, would soon convince him of his error.

---

**Renal Diabetes.** D. Sclater Lewis and Herman O. Mosenthal, Bull. Johns Hopkins Hosp., May, 1916. This condition consists in an abnormal permeability of the kidney to sugar, while the blood retains the normal level of 0.7-1.1 per mille. There is no loss of power to utilize carbohydrate and hyperglycaemia is not found. Hence, an increase in carbohydrate does not materially affect the discharge of sugar in the urine, the general symptomatology of diabetes melitus is lacking, the general health remains good and no tendency has been noted to the development of true diabetes. The term renal diabetes is objectionable and it should be described as renal glycosuria. Very few cases have been reported and some consider that the condition has not been definitely established. Tachau's observations are cited as follows: 4 days, carbohydrate-free diet, no urinary glucose; 6 days, mixed diet, 158-250 grams of carbohydrate, none; 15 days, mixed diet, 124-412 grams of carbohydrate, 1.1-6 grams of urinary glucose a day; 3 days, mixed diet plus 100 grams dextrose, 0.6-4.2 grams urinary glucose a day. (Days not in chronologic order). Blood sugar, fasting, .85 and .86 per mille; after breakfast .61 per mille; 1 hour after 100 grams of dextrose, .76, 1.09, .82 per mille. The authors' case was observed from Sept. 29-30 to Oct. 25-6. Two days of light ward diet (contents not specified) showed a urinary output of 13.1 and 11.1 grams of sugar a day, blood 1.3 per mille (too high), standard not accurate). 5 days of starvation diet reduced the urinary sugar from 11.9 to 0.7 and 2.1 grams per day, two blood examinations showing 1.1 per mille of sugar (too high) but acetone and diacetic acid appeared later remaining till the 18th day when the carbohydrate had been gradually increased to 28 grams, acetone alone remaining till the 20th day when the carbohydrate had been increased to 126 grams. Meantime, the urinary sugar had increased from 0 to 23 grams, several blood examinations, considered accurate being .9 per mille. For the remainder of the period of observation, the carbohydrate varied from 270 to 60, including glucose with meals, the urine remained free from acetone and diacetic acid but the urinary sugar varied from 11.5 to 30 grams a day, the blood on several examinations showing .9 and .8 per mille of sugar.

